# Healthy ageing: Herpes zoster infection and the role of zoster vaccination

**DOI:** 10.1038/s41541-023-00757-0

**Published:** 2023-11-28

**Authors:** Desmond Curran, T. Mark Doherty, Nicolas Lecrenier, Thomas Breuer

**Affiliations:** 1 GSK, Wavre, Belgium; 2 GSK, Tres Cantos, Spain

**Keywords:** Diseases, Drug discovery

## Abstract

Populations are ageing worldwide, with considerable time lived in ill-health, putting upwards pressure on healthcare budgets. Healthy ageing is defined as maintaining functional ability, including the ability to: meet basic needs; learn, grow and make decisions; be mobile; build and maintain relationships; and contribute to society. The risk and impact of infectious diseases increase with age due to immunosenescence. Vaccination can help to prevent disease in older adults, promoting healthy ageing and active lives. Herpes zoster (HZ) occurs when the varicella zoster virus is reactivated due to declining immunity. HZ is common, with a lifetime risk of one-third, and increases in incidence with age. HZ is associated with severe and intense pain, substantially affecting the functional status of patients as well as their overall health-related quality of life. HZ and its complications may result in prolonged morbidity, including persistent pain (post-herpetic neuralgia, PHN), hearing impairment, vision loss and increased risk of stroke and myocardial infarction. HZ and PHN are difficult to treat, substantiating the benefits of prevention. Vaccines to prevent HZ include a recombinant zoster vaccine (RZV). RZV has shown efficacy against the HZ burden of disease and HZ burden of interference on activities of daily living of over 90% in immunocompetent adults aged ≥50 years. Vaccine efficacy against HZ was maintained at over 70% at 10 years post-vaccination. Adult vaccination, including against HZ, has the potential to reduce burden of disease, thus helping to maintain functioning and quality of life to support healthy ageing in older adults.

## Introduction

Population ageing is a key demographic trend across developed countries globally. In the European Union, life expectancy has increased from 75 years in 1990 to 81 years in 2018, and is predicted to increase further by 2060^1^. In Japan, life expectancy at birth increased from 59.2 years in 1950 to 84.8 years in 2020^2^. By 2050, one person in six worldwide and one person in four in North America and Europe will be aged ≥65 years^3^.

While life expectancy is rising, considerable time spans are lived in ill health. For example, in 2017 men and women in Singapore were likely to spend the last eight and ten years of life, respectively, in poor health^4^. Healthcare utilisation tends to increase towards the end of life, with unpredictable and wide variation in the course of disability in the final years^4^. Disability and comorbidity, rather than age itself, are associated with increased healthcare expenditure. Consequently, maintaining good health in ageing individuals may limit increases in healthcare expenditure^5^. Maintaining function is key to improving quality of life and preventing the debilitating effects of immobility and inactivity in older adults^6^.

The prevalence and severity of many infectious diseases increase with increasing age. As well as the immediate effects of an acute episode of infectious disease, many older adults do not fully recover and may experience consequences such as exacerbation of chronic conditions, onset or worsening of frailty, difficulties with activities of daily living and loss of independence^7^. Vaccine-preventable diseases remain a major public health issue in older adults^8^. For example, herpes zoster (HZ), pertussis, pneumococcal disease and influenza can all result in reduced quality of life, activity and functioning in older adults, as well as a risk of mortality^6^. As a result of population ageing and population growth, the annual economic burden of these four diseases in adults aged ≥50 years in the United States (US) is projected to increase from US$35 billion to US$49 billion over the next 30 years^9^. More recently, coronavirus disease 2019 (COVID-19) has emerged as another vaccine-preventable illness disproportionately affecting older adults^10^.

HZ is a common and painful condition that occurs due to reactivation of varicella zoster virus (VZV) as a result of a decline in immunity. The incidence of HZ increases with age, together with the risk of complications such as post-herpetic neuralgia (PHN)^11^. Population ageing is projected to result in a substantial increase in the number of HZ cases in developed countries^11^.

Vaccines to prevent HZ approved for use in older adults include live-attenuated VZV vaccine (zoster vaccine live, ZVL) and an adjuvanted recombinant zoster vaccine (RZV)^3^. The potential contribution of HZ vaccination with ZVL to healthy ageing has been discussed in a previously published review^5^, and therefore the present review focusses on the RZV vaccine. The objectives of this review are to describe the burden of HZ and how vaccination with RZV has the potential to reduce both the risk and severity of disease, thus maintaining functioning and quality of life and supporting healthy ageing in older adults.

## Healthy ageing

Healthy ageing is defined as developing and maintaining functional ability to enable well-being in later years. Functional ability refers to the capabilities that allow a person to be and do what they value, including the abilities to: meet their basic needs; learn, grow and make decisions; be mobile; build and maintain relationships; and contribute to society^12^.

### Ageing and impact on Healthcare

The World Health Organization (WHO) Decade of Healthy Ageing report projects that the worldwide number of older adults aged ≥60 years will increase by 34%, from 1 billion in 2019 to 1.4 billion in 2030^13^.

**Fig. 1:**
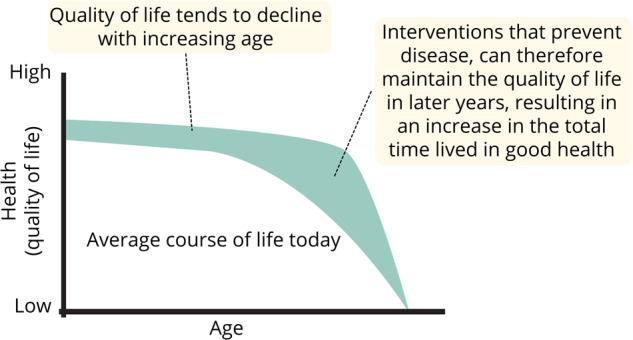
Schematic illustration of the potential benefit of healthy ageing, increasing the time lived in good health without extending life expectancy. Adapted from McKinsey 2022^83^.

The focus of most healthcare interventions has historically been on adding years to life, and the increase in life expectancy observed across the world in recent decades and projected for the future is an indication of the success of this approach. Healthy ageing focusses on adding quality to these extra years of life^14^. This is illustrated schematically in Fig. 1. In the current situation, average quality of life and functioning tends to decline with advancing age. The concept of healthy ageing concentrates on maintaining quality of life at a higher level for longer (Fig. 1). Even without further increases in longevity (both curves in Fig. 1 reach the X-axis at the same point), this has the effect of increasing the total time lived in good health (shaded area in Fig. 1).

Healthy ageing offers several potential benefits. In addition to the personal benefit of improved quality of life, older adults living additional years in good health can strengthen their societies by continuing to participate as integral parts of families and communities and strengthen healthcare systems by reducing the burden on the healthcare system^13^. There may also be economic benefits in relation to savings in healthcare costs associated with improved health and functioning, as interventions can be cost-effective or cost-saving^5^.

### Immune System and Ageing

Immunosenescence is the gradual deterioration of the immune system, brought on by natural age advancement^15^. It is associated with an increased susceptibility to infectious pathogens and may result in diminished response to vaccines in older adults^15,16^.

Ageing is associated with a chronic sterile inflammatory state called inflammageing^15^. This low-grade inflammation is predictive of frailty and earlier mortality, is a risk factor for some chronic degenerative diseases, and may be associated with age-related impaired humoral and cellular immune response to vaccination^15^. Many of the mechanisms involved in inflammageing are also involved in response to infection, and it has been suggested that exposure to infectious agents may promote biological reactions such as inflammation that accelerate ageing^17,18^.

### Impact of vaccination on healthy ageing

The burden of infectious disease is now concentrated in older adults^19,20^, and more adults than children die from vaccine-preventable disease^8,14^. The threat of infectious disease to ageing populations, and the gaps in the systems designed to address these risks have been highlighted in the COVID-19 pandemic, which may help to create a sense of urgency in developing adult immunisations and delivery systems^21^. Prevention of infectious disease by vaccination is part of a triad of interventions to promote healthy ageing, along with healthy diet and exercise^19^. The immune system retains some plasticity into older age, despite immunosenescence, and it has been suggested that vaccination may contribute to immune fitness, defined as the ability to mount an appropriate immune response to external challenges^22^. Immune fitness may be seen as a general state that reduces the risk of ill health, and is influenced by genetic factors and external factors such as diet, exercise, presence of chronic infection and avoidance of pollutants such as smoking. Vaccination may also be able to modulate the immune system, contributing to immune fitness in ways that are only beginning to be explored^22^.

Vaccination as a preventive strategy should span the life course, including adults and older adults as well as children^23^, and life-course vaccination could be a key tool for supporting healthy ageing^22^. For decades, most countries have implemented robust vaccination programme for children, but adult immunisation programme have consistently been a lower priority^17,19^, and vaccine coverage in older adults is lower than for children^6,19^. Reducing this vaccination gap is important to promote healthy ageing^6^.

Several vaccines are recommended for older adults, including vaccination against HZ, influenza and pneumococcal disease^7^. Vaccination in older adults offers substantial health and quality of life benefits^17^. In the US, for example vaccination programmes against HZ, influenza, pertussis and pneumococcal disease were estimated to avoid 65 million cases of disease over a 30-year period^24^.

## Herpes zoster

VZV lies dormant in nerve cells after a chickenpox infection, and in later life it can reactivate due to age-related immune system decline to cause HZ, also called shingles^3^. VZV infection is almost universal, and the lifetime risk of HZ is approximately one-third in the US and Asia-Pacific^25,26^. HZ is common in the US, with over one million cases a year^3^. A synthesis of HZ incidence rates reported in 61 records from 59 studies worldwide was carried out using meta-regression methods^27^. HZ incidence increased with increasing age, was higher in females than males, and increased over time^27^. It was estimated that 14.9 million cases of HZ occurred worldwide in people aged ≥50 years in 2020 and was projected to increase to 17.0 million in 2025 and 19.1 million cases by 2030, due to ageing of populations worldwide^27^.

The most common complication of HZ is PHN; it has been reported that pain lasting for 90 days or more following HZ rash onset occurs in 10–20% of HZ cases^28^. The risk of PHN increases with age and although the pain of PHN resolves within a year in the majority of cases, in some it may persist for years^28^. PHN in older individuals can have a long-term impact on the ability to carry out activities of daily living and can compromise functional status and result in loss of independence^3^. Other complications of HZ may also occur, including neurological complications such as palsies and stroke, eye complications such as iritis and vision loss, skin complications such as secondary bacterial infection, systemic complications such as pneumonia and myocardial infarction, and zoster-related hospitalisation or death^29^. HZ ophthalmicus can result in eye damage, pain, light sensitivity and loss of vision^30^. Symptoms of HZ oticus or Ramsay Hunt syndrome include tinnitus (48% of patients) and unilateral hearing loss (24% of patients), and hearing impairment may be permanent in 5% of cases^31^.

**Fig. 2:**
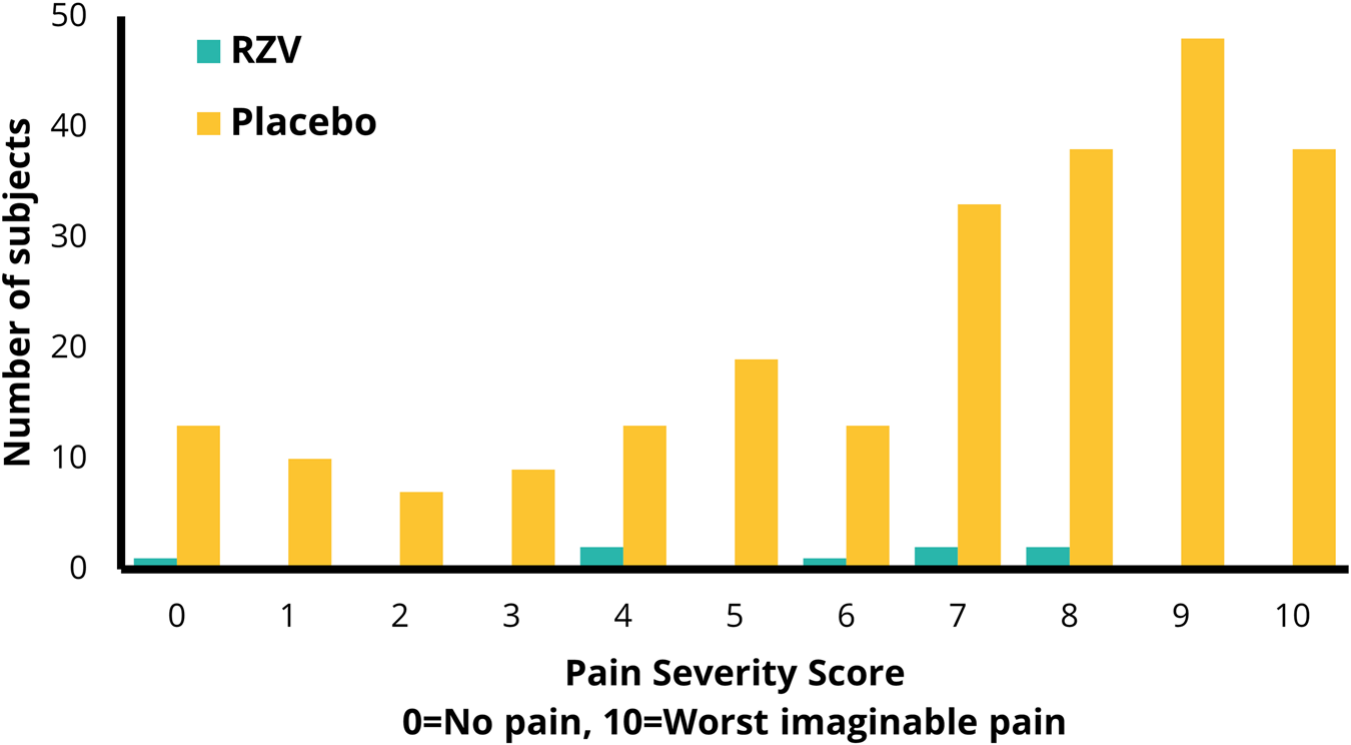
Maximum Zoster Brief Pain Inventory (ZBPI) worst pain severity scores by vaccination group in subjects ≥ 50 years of age. Based on data from Curran et al 2019^33^ with permission from Curran D. RZV, recombinant zoster vaccine.

HZ is characterised by localised rash and pain, with itchiness and fatigue also commonly reported^32^. The pain is frequently reported as severe and intense, with patients choosing language such as a blowtorch or an electric shock to describe their experience of the pain^32^. In the placebo group of a RZV vaccine trial in individuals aged ≥50 years, 65.2% of patients reported having ‘severe’ HZ pain (Zoster Brief Pain Inventory [ZBPI] score of 7 or above), and 15.8% reported ‘worst pain imaginable’ (ZBPI score of 10) (Fig. 2)^33^. In a *post-hoc* analysis of data from the placebo arms of three Phase III trials, increased ZBPI pain scores were significantly associated with worse scores across all domains of quality of life including physical, emotional and social impacts^34^. For every 1-point increase in ZBPI pain score, a 1.57-point decrease in SF-12 physical score is observed, demonstrating that HZ pain is associated with a loss of physical functioning^35^.

HZ-associated pain is frequently accompanied by discomfort such as pruritus (itching) and allodynia (pain caused by stimuli that would not normally be painful, such as touch, contact with hot or cold water, or air blowing on the skin)^36,37^. Allodynia can have a considerable impact on patients’ quality of life, causing difficulties with activities such as bathing, grooming and getting dressed and impairing social activities through fear of pain from being touched by others^32^. The negative effects of HZ/PHN on quality of life are broad-ranging, affecting multiple domains of quality of life, including impacts on sleep, mood, physical functioning, mental health, ability to undertake activities of daily living, work, and ability to participate in hobbies and social activities^28,32,38–43^. In a study in Germany, patients said their lives stopped due to the disease, and expressed feelings of helplessness, frustration, depression, sadness or rage, and they never forgot the illness and its impact on their lives^44^.

In a time trade-off study, individuals were willing to trade a mean of 89 discounted days to avoid the least severe scenario of HZ and a mean of 162 discounted days to avoid the most severe scenario, indicating that individuals place a substantial value on avoiding HZ^45^. As such there is an apparent paradox that many decision-making bodies on vaccination focus primarily on vaccines that prolong life, whereas individuals are willing to trade off time to avoid disease, thus maintaining their current quality of life. Consequently, vaccines that maintain quality of life should be given equal priority to those that extend length of life.

HZ and PHN are associated with a substantial economic burden in healthcare costs and productivity losses. Approximately 1–4% of patients with HZ require hospitalisation^46^, and patients with PHN reported an average of 9.5 consultations with healthcare professionals^28^. In the United Kingdom (UK), two-thirds of patients with HZ who were in employment reported that the condition had affected their work, mainly by having to take time off work^38^. Similarly, in a Canadian study, 64% of employed subjects reported missing work due to HZ (absenteeism), and 76% reported reduced effectiveness at work (presenteeism)^47^. Pain severity was associated with higher productivity loss^47^, and the presence of significant pain in HZ patients was associated with higher productivity losses compared with patients without pain^48^.

HZ and its complications may result in prolonged morbidity, as HZ is associated with a transient increased subsequent risk of stroke in the immediate period after the HZ episode^19^. The risk of stroke may be greater for HZ ophthalmicus than for HZ at other sites^49^. There may also be an increased risk of myocardial infarction, with a greater burden of disease in older adults with zoster^17^. The mechanisms are unknown, but may include direct effects on blood vessel walls causing inflammation^17^.

The pain from HZ and PHN is difficult to manage, with significant unmet need in treatment effectiveness and consequent dissatisfaction with treatment among HZ patients, which is presumably driven by experience of inadequate relief of symptoms^28,38^. Patients with PHN in the UK received multiple medications^28^, increasing the potential risks of polypharmacy in frail individuals. Given the challenges associated with managing HZ and PHN, preventative strategies for HZ and associated complications should be considered as a means of enabling patients to remain active in old age, minimising the individual patient and societal burden associated with the condition^28^.

## Recombinant zoster vaccine

Two classes of HZ vaccines are available for older adults, ZVL and RZV^3^. ZVL is a 1-dose vaccine that utilises the vOka live attenuated virus produced by serial passage of a wild-type clinical isolate termed pOka in human and guinea pig cell lines^50^. It is the same vaccine that is used to prevent varicella in children although with a higher potency (e.g. >14-times the varicella vaccine)^5^. ZVL is contraindicated in individuals with primary or acquired immunodeficiency^5^. RZV is a 2-dose non-live recombinant vaccine that combines the VZV glycoprotein E (gE) and the AS01B adjuvant system that helps to improve immunogenicity, especially in older adults, and permits vaccination of immunocompromised individuals^46^. AS01B is a liposome-based adjuvant comprising 3-*O*-desacyl-4′-monophosphoryl lipid A (MPL), a Toll-like receptor 4 ligand and QS-21, a saponin extracted from the bark of the *Quillaja saponaria* Molina tree (50 mg MPL and 50 µg QS-21). RZV has shown higher interleukin-2 and immune memory response than ZVL^51,52^. The second RZV dose should typically be given 2–6 months after the first; for persons who are or will be immunodeficient or immunosuppressed and who would benefit from a shorter vaccination schedule, the second dose can be administered 1–2 months after the first^53^.

Some countries, such as Canada and the UK, have preferential recommendations for RZV over ZVL, based on its high efficacy, long duration of protection and greater cost-effectiveness^54^. Parikh et al., in their overview of national vaccination recommendations for RZV, report much variation in age group recommendations, reflecting evaluations dependent on public funding, and differences with respect to use in immunocompromised and other special populations^54^.

ZVL has already been the subject of a comprehensive review^5^, and the present review discusses the RZV vaccine. Table 1 summarises published vaccine efficacy data for RZV. The vaccine efficacy of RZV against HZ was over 90% in immunocompetent adults aged ≥50 years in two phase III trials, ZOE-50 and ZOE-70^55,56^.

**Table 1:** Vaccine efficacy of RZV.

Vaccine efficacy against:	Vaccine efficacy	95% CI	Reference
HZ in immunocompetent adults aged 50+ years	97.2%	93.7, 99.0	^55^
HZ in immunocompetent adults aged 70+ years	91.3%	86.8, 94.5	^56^
PHN in immunocompetent adults aged 70+ years	88.8%	68.7, 97.1	^56^
HZ in immunocompetent adults aged 50+, year 10 post-vaccination^a^	73.2%	46.9, 87.6	^65^
HZ burden of illness (i.e. pain) in immunocompetent adults aged 50+ years	98.4%	92.2, 100	^33^
HZ burden of illness in immunocompetent adults aged 70+ years	92.1%	90.4, 93.8	^33^
HZ burden of interference with activities of daily living in immunocompetent adults aged 50+ years	99.1%	86.2, 100	^33^
HZ burden of interference with activities of daily living in immunocompetent adults aged 70+ years	90.3%	88.5, 92.1	^33^
HZ in frail individuals	90.2%	75.4, 97.0	^62^
HZ in pre-frail individuals	90.4%	84.4, 94.4	^62^
HZ in HSCT recipients	68.2%	55.6, 77.5	^63^
HZ in patients with haematological malignancies	87.2%	44.3, 98.6	^64^
HZ burden of illness in HSCT recipients	82.5%	73.6, 91.4	^74^
HZ burden of interference with activities of daily living in HSCT recipients	82.8%	73.3, 92.3	^74^

*CI* confidence interval, *HSCT* hematopoietic stem cell transplantation, *HZ* herpes zoster; *PHN* post-herpetic neuralgia, *RZV* recombinant zoster vaccine.

^a^Data accrual for year 10 is still ongoing^65^.

Efficacy was independent of age, sex or ethnicity^57^, and was maintained in patients with common pre-existing medical conditions such as hypertension and diabetes^58–61^. Vaccine efficacy was similar in subjects aged 70–79 years and in subjects aged ≥80 years^56^, indicating that RZV is able to overcome immunosenescence. Furthermore, in a *post-hoc* analysis of pooled data from the ZOE studies, vaccine efficacy was above 90% in frail, pre-frail and non-frail subgroups^62^. RZV has also demonstrated efficacy against HZ in phase III trials of immunocompromised adults aged ≥18 years 1) who had recently undergone autologous HSCT^63^, and 2) with haematological malignancies who had recently undergone immunosuppressive cancer treatments (a *post-hoc* analysis)^64^.

An interim analysis of data collected with up to 10 years follow-up after vaccination in the ZOE-50 and ZOE-70 trials estimated vaccine efficacy at 84.2% for year 8, 72.7% for year 9 and 73.2% for year 10 post-vaccination^65^. Data accrual beyond year 10 is still ongoing and will be reported in a final analysis^65^. By reducing HZ, RZV also protected against PHN^56^.

No safety concerns have been identified with RZV, although transient injection site and systemic reactions were more common than with placebo. Most reactions were mild to moderate in intensity and transient (median duration of 1–3 days)^55,56^. Second dose completion rates of RZV were approximately 95% in clinical trials and approximately 70% to 80% in real world studies, similar to what has been observed for other adult vaccines suggesting vaccine reactions is one of many factors that influence completion rates^55,56,66–68^. Except for these reactions, the safety profile of RZV was comparable with placebo, regardless of age, gender or race^69,70^. A self-controlled case series analysis indicated a slight increase in the risk of Guillain-Barré syndrome in the 6 weeks after vaccination (approximately 3 excess cases per million vaccinations); the risk-benefit balance remained in favour of vaccination^71^. The available information is insufficient to determine a causal relationship^72^. The safety profile of RZV remained clinically acceptable at year 10 post-vaccination^65^.

Data from the ZOE-70 study indicated that RZV significantly reduced HZ-associated pain medication use and duration of pain medication use in participants with confirmed HZ^73^. Figure 2 presents the distribution of the individual maximal ZBPI “worst-pain” scores experienced over the entire HZ episode for the ZOE-50 study^33^. In addition to preventing HZ episodes (and the pain associated with those episodes), RZV also attenuated the severity of pain in individuals with breakthrough disease^33,74^. More importantly from a healthy ageing perspective, RZV reduced the burden of HZ pain and the impact of HZ on activities of daily living by greater than 90% in adults aged ≥50 years^33^. Both “burden of illness” and “burden of interference” were composite endpoints incorporating incidence, duration, and severity (or interference) of HZ.

Modelling studies have indicated that RZV could reduce projected healthcare resource use, such as hospitalisations, outpatient visits and general practitioner visits^75,76^, and reduce work losses due to HZ^77^. Vaccination with RZV was reported to be cost-effective in 15 of 18 studies in a recent review, and cost-effective or cost-saving compared with ZVL in the subset of these studies where a comparison to ZVL was made^78^. In a modelling study in Germany, based on long-term efficacy data up to 8 years post-vaccination, the number needed to vaccinate (NNV) with RZV to prevent one HZ case was estimated at 6–10, and to prevent one PHN case it was 34–48^79^. The NNV to prevent one HZ case was 6 for individuals aged 50–69 years of age compared with 10 in individuals aged ≥70 years of age^79^. As such, vaccinating individuals who are younger (and potentially healthier) may provide the best public health impact, given the long-term efficacy observed for the vaccine. Vaccination of healthy individuals ensures that individuals are vaccinated when the immune response is likely to be most robust.

Recent reports suggest that HZ vaccination may reduce the risk of dementia^80,81^. These findings were based on analyses of retrospective databases where matched cohorts of vaccinated and unvaccinated individuals were compared. Further research is required to 1) better understand the mechanisms that may lead to this association and 2) confirm these associations via prospective clinical studies.

## Summary and conclusion

**Fig. 3:**
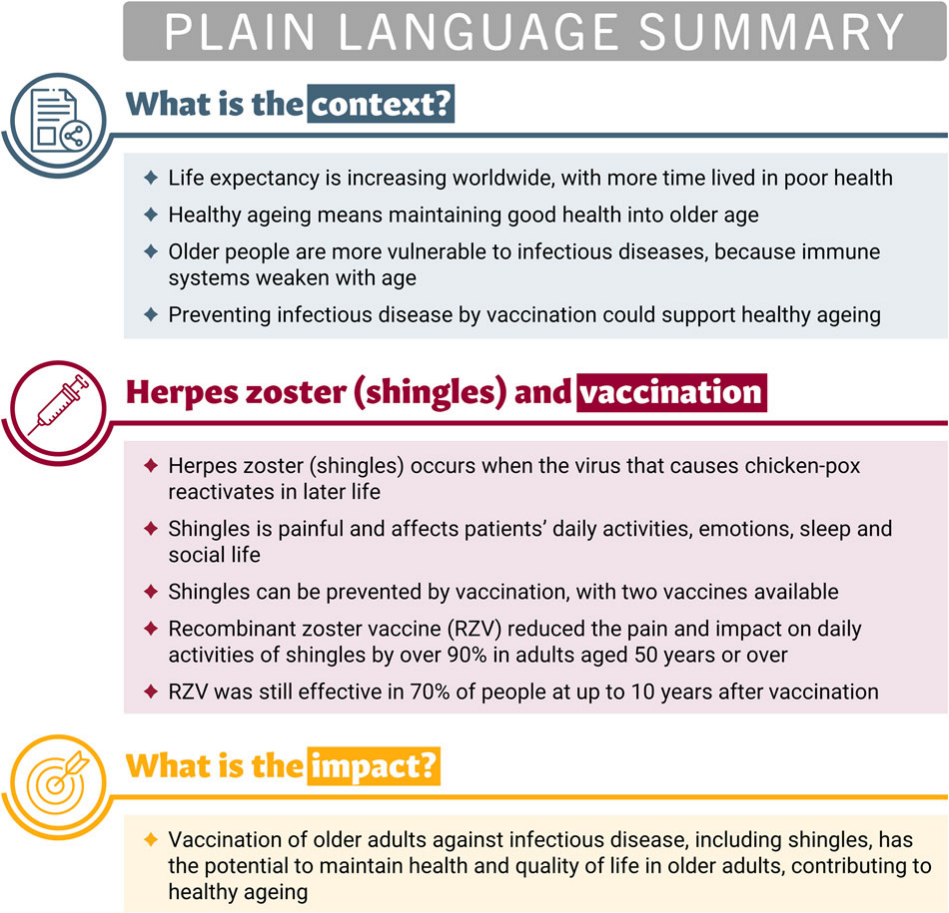
Plain language summary.

**Fig. 4:**
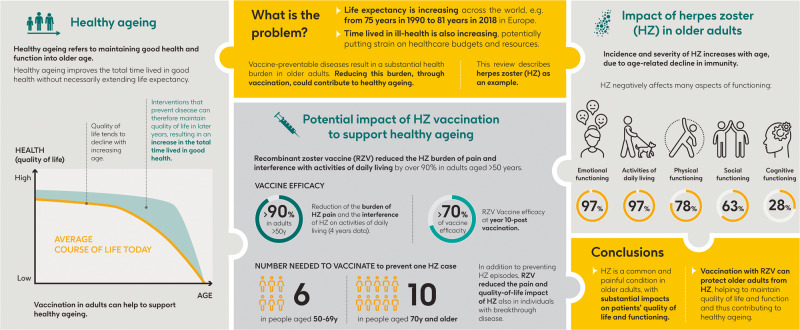
Graphical abstract. y years.

A plain language summary of our findings and their relevance is presented in Fig. 3. The main findings are also represented visually in a graphical abstract in Fig. 4. Population ageing is an established demographic trend worldwide^3^. With older adults making up an increasing proportion of the population, age-related increases in disability and morbidity have the potential to overburden healthcare budgets and systems. Healthy ageing is defined as developing and maintaining functional ability to enable well-being in later years^12^. Health interventions need to focus on improving the quality of these longer lifespans, promoting healthy ageing that maintains good health and function into older age.

Immune function characteristically declines with age, resulting in higher prevalence and severity of infectious disease. The burden of vaccine-preventable infectious diseases is now higher in adults rather than children in developed countries such as the US^20^, due to established vaccination programmes for children with overall reasonably high vaccination rates. However, adult vaccination is less established with much poorer coverage than childhood vaccination^20^.

HZ and complications such as PHN, HZ ophthalmicus and HZ oticus occur mainly in older adults. HZ and its complications are associated with prolonged morbidity such as vision loss, hearing impairment, increased risk of stroke, impaired functional status and loss of health-related quality of life^3,19,30,31^.

In addition, a substantial burden in healthcare costs such as hospitalisation^46^ and lost work time^47^ is observed for affected individuals. HZ and PHN are difficult to manage with current treatments^28^. Prevention of HZ through adult vaccination could therefore offer substantial benefits.

RZV has been shown to be effective in reducing HZ incidence^55,56^, the HZ burden of illness (i.e. pain), and the interference of HZ with activities of daily living in older adults^33^. Modelling studies also indicate that RZV would be expected to substantially reduce healthcare visits and hospitalisations^75,76^, and reduce work losses due to HZ^77^. As social connections and relationships contribute to the life satisfaction of individuals^82^, reducing the negative effects of HZ on social functioning offers the potential to improve life satisfaction and well-being in older adults.

Adult vaccination programmes, including against HZ, have the potential to reduce morbidity in older adults, maintaining quality of life and providing important social and economic benefits. Adult vaccination is a key component of healthy ageing. The goal for adult vaccination programmes should be to reach the level of maturity currently observed for paediatric vaccines, with the necessary infrastructure to achieve high coverage. This would contribute towards promoting healthy ageing, adding not only years to life but more importantly enhancing the quality of those remaining years.

## Data Availability

All data included in this review were obtained from publicly available sources and cited accordingly. The references for each study or publication included in this review are provided for further reading and verification. No additional datasets were generated or analyzed specifically for this manuscript.
